# Pilot Study to Estimate Dietary Fiber Intake in Adults Residing in Chile

**DOI:** 10.3390/nu15040900

**Published:** 2023-02-10

**Authors:** Carla Guzmán, Jonathan Espinoza, Fabiola Fuentealba

**Affiliations:** 1 School of Nutrition and Dietetics, Faculty of Health Care Sciences, Universidad San Sebastián, Campus Concepción, Concepción 1457, Chile; 2 Vice Rectors Office for University Outreach, Universidad San Sebastián, Concepción 1457, Chile

**Keywords:** dietary fiber, food frequency questionnaire, questionnaire screening, validation

## Abstract

Dietary fiber has been associated with health benefits; therefore, the availability of validated tools to assess food consumption associated with high-fiber foods would allow the quantification of the intake of this functional nutrient, the identification of risk groups and target populations, and the development of public policies and/or programs aimed at improving the health of the population. In this study, a fiber intake short food frequency questionnaire (FFQ) was translated into Spanish, and its content validity was determined by a group of experts, to subsequently conduct a pilot test including 198 subjects aged 36 ± 12.5 years, residing in Chile (46 men and 150 women), with the purpose of quantifying dietary fiber intake. The global assessment of the FFQ revealed a validity coefficient of 0.98 ± 0.02; after the application of the pilot, the mean dietary fiber intake in adult Chilean residents was 12.3 g per day, with similar results to those found in the National Food Consumption Survey 2010 (12.5 g per day in men, and 11.5 g in women). The FFQ is a quick and valid tool to classify people on the basis of their habitual dietary fiber intake.

## 1. Introduction

**Figure 1:**
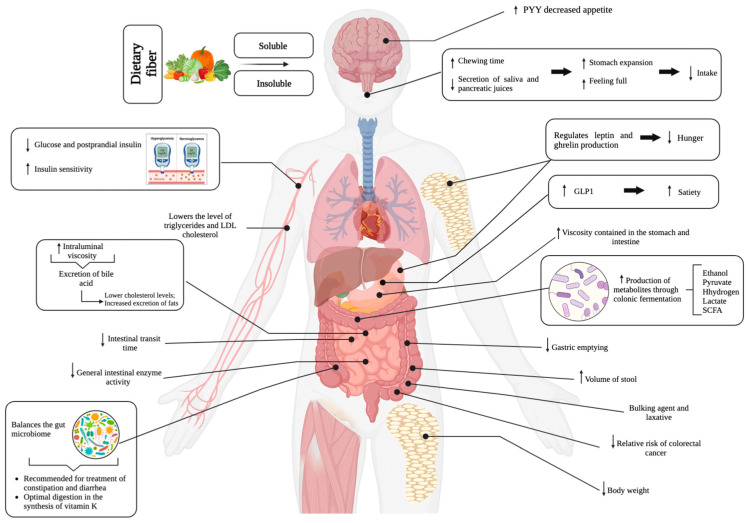
Benefits of dietary fiber consumption on human health. PYY: peptide YY; GLP1: glucagon-like peptide 1; SCFA: short-chain fatty acid. ↑: increase; ↓: decreases.

Several definitions of dietary fiber (DF) have been proposed over time, based primarily on the physiological aspects or on the methods used for its analysis, as defined by the AOAC (Association of Agricultural Official Chemists) [1]. DF includes a diverse range of complex carbohydrates that play a critical role in public health, since this is an under-consumed nutrient, when compared to the recommended intake in most countries worldwide [2]. The codex alimentarius states that dietary fiber means carbohydrate polymers with ten or more monomeric units, which are not hydrolyzed by the endogenous enzymes in the small intestine of humans and belong to the following categories: (a) edible carbohydrate polymers naturally occurring in the food as consumed; (b) carbohydrate polymers, which have been obtained from food raw material by physical, enzymatic, or chemical means and which have been shown to have a physiological effect of benefit to health as demonstrated by generally accepted scientific evidence to the competent authorities; and (c) synthetic carbohydrate polymers which have been shown to have a physiological effect of benefit to health as demonstrated by generally accepted scientific evidence to the competent authorities [3]. On the other hand, DF can be classified into soluble dietary fiber (SDF) and insoluble dietary fiber (IDF) according to its ability to hydrate in water [4]. The food sources of SDF are apples, pears, citrus fruits, carrots, broccoli, peas, cucumber, celery, and wheat bran [5]. SDF forms colloid solutions in the intestine, slows digestion, and causes a prolonged feeling of satiety, while IDF accelerates intestinal transit, increases stool volume, and acts as a bulking agent and laxative. Its food sources are nuts, legumes, whole wheat, barley, and tubers [6]. Considering the above, DF is related to the promotion of the mitigating effects on cholesterol and glucose levels [7,8], with the latter being related to the onset of type 2 diabetes [9,10], obesity, colon cancer [11,12], and cardiovascular diseases [13,14]; it takes part in all of the functions of the digestive system, from mastication to the evacuation of feces [15], and in general, health benefits provided by DF include improvements in gut health (increase in fecal loading, softening of feces, decrease in fecal pH, and fermentation) [16], glycemic and insulinemic control, cholesterol reduction (total cholesterol and LDL-cholesterol) [9], weight control (reduction of caloric intake and increase in satiety) [17], and an effect on the metabolic function of the different microbial species that colonize the gastrointestinal tract, to improve human health and potentially prevent or treat diseases in general [18]. The beneficial effects associated with DF consumption are shown in Figure 1.

Generally speaking, the daily intake of DF should be in the range of 18–38 g per day for adult subjects [15]; nevertheless, DF intake does not exceed 20 g per day at the global level [19]. Against this background, the Scientific Advisory Committee on Nutrition in the UK recommended in 2015 to increase the intake to 30 g per day; however, only 9% of adults in the UK managed to reach this objective [20], while in the United States, according to data from the National Health and Nutrition Examination Survey (NHANES) 2009–2010, mean fiber intake was 16.2 g per day, representing an increase of approximately 1 g per day with respect to the 15.1 g per day reported in the 2001–2002 survey. Nevertheless, this intake continues to fall well short of the recommendations [19].

In Chile, there is scant information available regarding food consumption, especially on the quantification of fiber intake. The epidemiological profile of the country has changed dramatically in recent decades [21], implying significant modifications in the dietary profile; these changes need to be addressed. The last data correspond to those collected by the National Food Consumption Survey 2010 (ENCA, as its acronym in Spanish), that reported a low consumption of foods that contain protective nutrients, such as DF, showing an average intake of 12.5 g per day in men, and 11.5 g per day in women [21], which are below the recommendations suggested by the Joint FAO/WHO Expert Committee, whose advice indicates an average intake of 25 g per day [14]. In this regard, the availability of validated tools to assess food consumption, particularly associated with the intake of high-fiber foods, would allow the quantification of the intake of this functional nutrient, the identification of risk groups and target populations, and the development of public policies and/or programs aimed at improving the health of the population.

The objective of this research was (a) to adapt and validate the content of a dietary fiber intake short food frequency questionnaire through an assessment by experts; (b) to perform a pilot test of the dietary fiber intake short food frequency questionnaire in adults residing in Chile.

## 2. Materials and Methods

### 2.1. Food Frequency Questionnaire to Assess Dietary Fiber and Scoring Sheet

**Figure 2:**
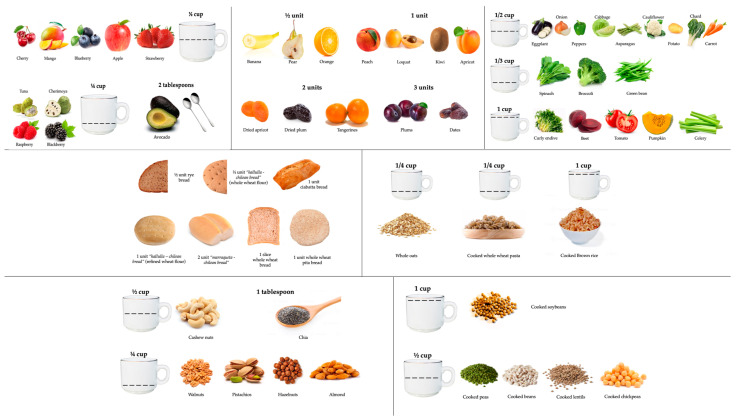
Representative images of each food group, according to the Photographic Atlas of Typical Chilean Preparations.

In the year 2022, we conducted the pilot phase of an observational study for the validation of the dietary fiber intake short food frequency questionnaire (DF-FFQ) developed by Healey et al. (2016), who authorized its use for the performance of the present study [22]; the questionnaire was implemented as an online instrument, because compared with traditional methods, online dietary assessment methods can be used to target specific geographical population groups, can be accessed remotely, and can be designed to be easy to complete [23]. The questionnaire includes 59 foods that contain fiber classified in 5 food groups (fruits, vegetables, bread-cereals, dried fruits, and pulses), with consumption frequencies ranging from “never” to “6 or more times per day” (Supplementary Materials S1). Considering the high consumption of bread in the country [21], it was decided to subdivide the cereal food group, estimating fiber intake independently from bread consumption. In addition, in order to reduce reporting bias related to food serving size, representative images of each food group were added, according to the Atlas Fotográfico de Preparaciones Típicas Chilenas [Photographic Atlas of Typical Chilean Preparations] [24]. The included food groups are presented in Figure 2.

Fiber content was calculated using the Table of Chemical Composition of Chilean Food [25], and a scoring sheet, which was similar to the original version, was developed to quantify the amount of dietary fiber consumed by each participant (Supplementary Materials S2). The total intake of dietary fiber of an individual was calculated as the sum of the average amount of fiber consumed from each food group in relation to the number of consumed servings.

### 2.2. Content Validity of Food in the DF-FFQ

**Figure 3:**
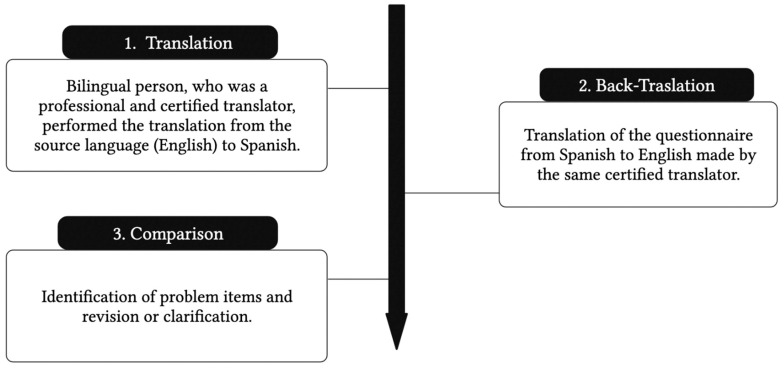
Translation process of the original questionnaire to its Spanish version.

The initial step in the process of validation of the instrument consisted of following the internationally suggested guidelines [26], that consider the translation of the questionnaire from the source language (English) to its Spanish version, with the translation being performed by a certified translator (Figure 3).

Subsequently, the instrument was presented to a commission of expert judges, composed of 6 scholars (dietitian-nutritionists) who work in the field of nutrition and food science on the basis of their professional experience, specialty, and academic degree [27]. Experts were separately given a closed-question survey to evaluate the adequacy and pertinence of the Spanish-translated version; the survey was based on a Likert-type scale including five alternatives (1 = strongly disagree; 2 = disagree; 3 = undecided; 4 = agree; 5 = strongly agree) (Supplementary Materials S3). Among the criteria examined were the clarity of wording, clarity of instructions, adequate language, whether it measures what it intends, and the extent to which it fulfills its stated purpose. They were also given the opportunity to add comments and/or suggestions, as in open-ended questions. Items that did not meet the minimum standards were reconsidered for modification or elimination. From this step, the final version of the DF-FFQ (dietary fiber food frequency questionnaire) was obtained. 

A content validity index (CVI): I-CVI—(item-level content validity index); acceptable limit >0.80 and S-CVI/Ave—scale-level with universal agreement method; acceptable limit >0.80 [28,29]. In addition, we used the method proposed by Hernández-Nieto 2002 [30], where each item of the instrument was verified by calculating the individual mean value (acceptable limit > 0.80), based on the following formula CVC (content validity coefficient):

CVC = (M_x_/V_máx_) − Pe_i_ (1)

where M_x_ represents the mean of the element in the score given by the experts, V_máx_ the maximum score that the item could reach, and Pe_i_ the error assigned to each item.

### 2.3. Determination of Dietary Fiber Intake Using the FFQ and Ethical Aspects

With respect to the application of the pilot test of the Spanish version of the questionnaire, participants consisted of adults aged 18 years and older, residents of Chile, and those with Internet access; the test was carried out from April to August 2022. All subjects who presented a condition that hindered the consumption of food sources with high-fiber content were excluded from the study (severe liver disease; kidney failure; gastric cancer and colon cancer; diagnosis of food allergies and food intolerance; celiac disease; Crohn’s disease; ulcerative colitis; pregnancy; and/or having a disease that causes a disruption in consumption patterns). The research team used a non-probabilistic sampling method, issuing an open call to community members through virtual/social platforms. The recruitment process involved the initial screening and obtaining the informed consent. A total of 198 participants were recruited, whose sociodemographic (sex (men or women), age (years), occupation (dependent worker, freelance worker, student, unemployed, housekeeper, or retired), place of residence (urban or rural)) and nutritional variables (weight (kg), height (m), body mass index (kg/m^2^)) were considered; in addition, subjects were given an FFQ to assess dietary fiber intake. The research was developed following the Declaration of Helsinki [31] regarding work involving human beings, and the CIOMS Guidelines [32] were also considered. The protocol of this study was approved by the Scientific Ethics Committee of the Universidad San Sebastian (No. 5–22). All participants were fully informed and expressed their willingness to participate by signing the informed consent form.

### 2.4. Statistical Analysis

A statistical analysis was performed with the IBM SPSS Statistics V21 software (Armonk, NY, 2012). The validation of the survey content was verified for each item of the questionnaire, indicating the percentage of agreement among the expert judges, with a preset level of S-CVI/Ave, S-CVI/UA, and CVC = 0.80 deemed acceptable. The descriptive statistics for quantitative variables were described with average and standard deviation, while categorical variables were described with frequencies and percentages. Variables were tested for normality using the Kolmogorov–Smirnov test, and the Mann–Whitney test was used to evaluate the differences between groups. A *p* value of < 0.05 was considered statistically significant for all tests. 

## 3. Results

A global assessment of the questionnaire demonstrated that the validity coefficient reached values higher than 0.8; specifically, I-CVI and S-CVI/Ave averaged 0.92 and 0.97, respectively. Additionally, adequacy averaged a CVC of 0.98, whereas pertinence averaged an adequacy CVC of 0.95, with no need to modify a particular item; nevertheless, it was decided to split the “cereals and bread” group up, because, considering the high consumption of bread among the Chilean population, it is worthy of a separate categorization. Detailed values for each item are shown in Table 1. Based on the opinions provided by external judges in the open-ended questions, the quality of some representative images of food items and their respective serving size were modified, to facilitate greater understanding of the questionnaire. 

The questionnaire was applied to 198 participants residing in the national territory. The sociodemographic variables and nutritional status are shown in Table 2. The mean age of the population was 36.0 ± 12.5 years; most of the subjects were Chilean citizens (94.2%); predominantly females (72.5%); concentrated mostly in the regions of Biobio, Maule, and Valparaíso; specifically in urban areas (85%), with respect to rural areas, which only reached 10.6%. The occupation of the participants, in decreasing order, corresponded to dependent worker (45.9%), students (26.1%), and independent worker (10.1%). When analyzing the overall data, it was observed that 64.6% of the surveyed population had excess weight, with 37.9% classified as overweight, and 26% classified as obesity (Table 2).

The mean dietary fiber intake values were 12.3 ± 6.9 g/d for the total population (Table 3). Average fiber intake and intake by food groups according to age, sex, residence, occupation, and BMI category are shown in Table 4. When comparing fiber intake by food groups and by age groups, a significant difference was observed in the cereal group (*p <* 0.001), with a higher consumption in people aged 15–29 years (2.87 g/d) versus ≥30 years of age (1.33 g/d), while for the sex variable, a statistically significant difference was only observed in the amount of fiber contributed by bread (*p =* 0.013), where men were those who consumed it in a greater quantity, surpassing women in 1.44 g/d of total fiber. Likewise, fiber intake according to age and occupation was statistically different only in the cereal group; however, when comparing the nutritional status, a difference was observed in the fruit, bread, and cereal groups, with a lower consumption of fiber in the participants with obesity, with respect to those with a deficient nutritional status.

**Figure 4:**
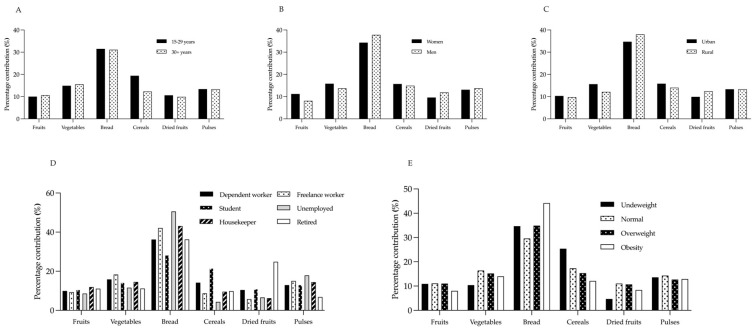
Percentage contribution to fiber intake (%) by food group in (**A**) age; (**B**) sex; (**C**) residence; (**D**) occupation; (**E**) BMI classification.

The contribution to total fiber intake by age, sex, residence, occupation, and BMI category for each food group is shown in Figure 4. When analyzing each variable individually, it was observed that fiber intake from bread consumption was predominant, followed in most cases by cereals. Overall, a greater contribution to total fiber intake was observed from bread (35.2%) and cereals (15.5%), while dried fruits (10.3%) and fruits (10.3%) were the items that contributed the least to total intake.

## 4. Discussion

To our knowledge, currently there are no short questionnaires for the quantification of dietary fiber intake in the Chilean population, which makes it difficult to classify users based on their dietary fiber intake and their association with different health events, whose etiology is partly related to the lower or higher intake of such food components.

This pilot study was intended to adapt the process of validation of the content of a dietary fiber intake short food frequency questionnaire, which was adapted from a previously validated English version [22], through an assessment by experts and the subsequent implementation of a pilot test with adults residing in Chile.

Content validity is one of the most important types of validity to ensure congruence between the study objective and data collection instrument; therefore, it is a common starting point for questionnaire validation [33]. As Lynn (1986) pointed out, researchers calculate two types of CVI [34]. The first involves the content validity of the items individually (I-CVI), while the second involves the content validity of the overall scale (S-CVI-Ave) [29]. Our results indicate that the DF-FFQ was considered by experts to have excellent clarity and relevance. The I-CVI and S-CVI-Ave values were above the minimum standard of 0.80, and the CVC for most items was rated as excellent in both adequacy and pertinence. Participants found the questionnaire to be acceptable and understandable, demonstrating the content validity of the questionnaire, with minor modifications suggested by the external judges, such as specifying the serving size of some food items.

Considering that in Chile there are no known short questionnaires for the quantification of dietary fiber intake, the performance of a pilot study is an essential step in the research process to quantify the intake of this nutrient in the population living in the national territory, since it allows the evaluation of the methodological and procedural aspects of a larger scale future study; therefore, its planning, implementation, and dissemination must be rigorously conducted in accordance with the guidelines proposed for pilot studies [35,36]. Moreover, the publication of this study is relevant, since its scope is to identify the adequacy of the instrument, and therefore achieve a greater degree of representativeness in the development of future research, including a larger sample size.

In total, 198 people residing in Chile participated in the pilot study, 94% of whom were Chilean citizens living in urban areas, with the Biobio, Maule, Valparaiso, and Metropolitan regions being reported as the most representative, which is in line with data on population density published in Chile [37]. The sample consisted predominantly of women, who represented 72.5% of the total population, similar to what was observed in other studies on dietary intake [22,38]. This is consistent with the demographic distribution of the country, where women represent more than half of the population [37].

Regarding DF intake, the average intake was 12,3 g per day, falling well short of the intake recommendation of this nutrient that should be in the range of 18–38 g per day for adult subjects [15], which is associated with health benefits such as cardiovascular health [39], blood glucose levels [8], gastrointestinal health [16], and obesity and weight management [40]. According to data previously published by the National Food Survey in Chile, the low consumption of total fiber has been maintained [21], which is a situation similar to other countries with insufficient consumption such as Brazil (15.7 g/d) [41], Argentina (9.3 g/d) [42], Mexico (16 to 18 g/d) [43], or the United States (18.3 g/d) [44], while studies in European countries such as Sweden and Norway established a total fiber intake of 19.6 g/d and 24.0 g/d, respectively [45].

With respect to fiber intake, according to age range, the results of the National Diet and Nutrition Survey in the UK (2014) and the Australian National Survey (2017) have shown that the intake of this nutrient increases with age, with a marked decrease after the age of 65 years [45,46]. However, in this research, the highest fiber intake was observed in the population aged 15–29 years, with a decrease of approximately 4 g in the intake of the population aged 30 years or older, which can be explained by differences in the classification of age groups, since there was no subclassification between adults and older adults.

On the other hand, our results show a significantly higher intake in the male population, probably explained by the higher consumption of bread and cereals in this population, which are the food sources with the highest contribution to the total intake of this nutrient. This is a situation that is repeated when compared with other studies, in which a higher average daily intake has been identified in men (17.3 g/d) than in women (14.9 g/d) [47]. Likewise, Brazilian researchers have affirmed that rural households had a higher fiber intake than urban ones [41], as observed in our research, which can be explained by the lower industrialization of food available in rural areas [48].

Participants in this study with an underweight body mass index (BMI) had an average total fiber intake of 24.2 g/d, while those participants with obesity reported an intake of 11.1 g/d. Therefore, it was shown that those with higher BMI consumed significantly less fiber. Several studies have shown that individuals with a higher total fiber intake experience less annual weight gain [49].

Another relevant finding reported in these preliminary results showed that bread made the greatest contribution to DF intake, representing 34.3% of total fiber intake.

This result is in accordance with the bread consumption pattern observed in Chile [21], since according to data reported by the Federación Nacional de Industriales Panaderos (National Federation of Bakers, FECHIPAN, as its Spanish acronym), Chile is the second largest consumer of bread worldwide after Turkey, with a consumption of 98 kg per capita [50]. Bread consumption is a deeply rooted habit in Chile. Studies carried out by the Oficina de Estudios y Políticas Agrarias (Office of Agrarian Studies and Policies, ODEPA, as its Spanish acronym), which investigated the perceptions of Chilean consumers, indicated that bread is considered basic and indispensable. However, most consumers do not attribute nutritional benefits to bread. However, this food can be excellent for a balanced diet [51]. Given the plethora of scientific evidence corroborating the multiple and varied health benefits of dietary fiber, and the risks associated with a diet that lacks fiber, the optimization of fiber within our diets represents an important public health strategy to improve both metabolic and overall health, and one of the possible strategies is to increase the fiber content of bread.

The survey showed a great practical relevance in the Chilean population, since the criterion to select food sources and the way of estimating dietary fiber contribution facilitated the use of the tool. This study has shown that the FFQ is a quick and valid tool to classify people based on their habitual dietary fiber intake. However, despite the success of the pilot test, the results may not be generalized nationwide because of methodological limitations, particularly sample size (as it is a pilot study), and the concentration of responses that corresponded mostly to the Biobio, Maule, and Metropolitan regions; therefore, in the future, it is expected that the development of a stratified questionnaire be applied at a national level, with the purpose of having a more accurate estimate of reality with respect to dietary fiber intake in the population residing in Chile.

## 5. Conclusions

This research showed that the Chilean population has a low consumption of dietary fiber, mainly among women and people with a higher body mass index. In this sense, it is important to promote the consumption of fiber, through messages that guide to better food selection, detailing the benefits it provides for the prevention of chronic diseases and the best quality of life of the population. Likewise, knowing the intake of nutrients and their dietary sources is crucial for the development of public health policies and behavioral change strategies to improve dietary intake.

## Figures and Tables

**Table 1:** Mean, standard deviation, confidence interval, and validity coefficient for adequacy and pertinence of the DF-FFQ instrument.

Variable	Item	Mean	SD	95% CI	CVC	S-CVI/Ave
Adequacy	1	4.9	0.15	4.73–5.06	0.98	1.00
	2	5.0	0.00	5.00–5.00	1.00	1.00
	3	4.8	0.28	4.53–5.13	0.96	1.00
	4	4.9	0.20	4.70–5.13	0.98	1.00
	5	4.9	0.12	4.82–5.07	0.98	1.00
	6 -	4.8 -	0.40 -	4.40–5.26 -	0.96 0.98	1.00 1.00
Pertinence	1	4.7	0.44	4.14–5.25	0.94	1.00
	2	4.7	0.44	4.14–5.25	0.94	1.00
	3	4.5	0.86	3.42–5.57	0.90	0.80
	4	5.0	0.00	5.00–5.00	1.00	1.00
	5	4.9	0.22	4.62–5.17	0.98	1.00
	6 -	4.7 -	0.44 -	4.14–5.25 -	0.94 0.95	0.80 0.93

Item 1: fruits; Item 2: vegetables; Item 3: bread; Item 4: cereals; Item 5: dried fruits; Item 6: pulses; SD: standard deviation; 95% CI: 95% confidence interval; CVC: content validity coefficient; S-CVI/Ave: scale-level content validity index based on the average method.

**Table 2:** Descriptive analysis of sociodemographic variables and nutritional status (*n* = 198).

Variables		Participants (*n*)	Percentage (%)
Age	15–29 years	76	38.4
	≥30 years	122	61.6
Sex	Men	46	22.2
	Women	150	72.5
	Prefer not to answer	2	1.0
Region	Antofagasta Region	1	0.5
	Valparaiso Region	30	14.5
	O’Higgins Region	1	0.5
	Maule Region	36	17.4
	Biobio Region	101	48.8
	Los Lagos Region	1	0.5
	Magallanes Region	1	0.5
	Metropolitan Region	23	11.1
	Ñuble Region	3	1.4
Residence	Urban	176	85.0
	Rural	22	10.6
Occupation	Dependent worker	95	45.9
	Freelance worker	21	10.1
	Student	54	26.1
	Unemployed	7	3.4
	Housekeeper	15	7.2
	Retired	6	2.9
BMI	Underweight	3	1.5
	Normal	68	34.5
	Overweight	74	37.6
	Obesity	52	26.4

BMI: body mass index (kg/m^2^).

**Table 3:** Description of total fiber intake (g/day) (*n* = 196).

Variable	Mean	SD	Minimum	Maximum
Age				
15–29 years	14.8	8.8	0.2	40.3
≥30 years	10.8	5.0	0.6	26.3
*p* value	0.004 *			
Sex				
Women	11.7	6.47	0.20	32.4
Men	14.4	8.33	3.00	40.3
*p* value	0.045 *			
Residence				
Urban	12.2	7.12	0.20	40.3
Rural	13.5	6.09	3.90	28.6
*p* value	0.233			
Occupation				
Dependent worker	11.4	5.35	0.60	26.3
Freelance worker	10.6	5.24	3.00	26.2
Student	15.6	9.65	0.20	40.3
Unemployed	12.2	6.48	3.50	21.9
Housekeeper	9.93	4.67	0.80	17.4
Retired	10.8	4.57	5.00	18.2
*p* value	0.181			
BMI category				
Underweight	24.2	5.56	17.9	28.5
Normal	13.7	8.15	3.90	40.3
Overweight	11.4	6.53	0.60	37.4
Obesity	11.1	5.01	0.20	22.2
*p* value	0.037 *			

Data are presented as mean, standard deviation (SD), minimum, and maximum. BMI: body mass index (kg/m^2^). Comparisons between categories were performed with Mann–Whitney U test or Kruskal–Wallis test as appropriate; (*) statistical significance *<* 0.05.

**Table 4:** Description of fiber intake (g/day) by food groups (*n* = 196).

	Fruits			Vegetables			Bread			Cereals			Dried Fruits			Pulses		
	Mean	SD	Range	Mean	SD	Range	Mean	SD	Range	Mean	SD	Range	Mean	SD	Range	Mean	SD	Range
Age																		
15–29 years	1.48	1.43	0–5.2	2.21	1.83	0–8.4	4.66	2.87	0–11.2	2.87	3.28	0–17.0	1.57	2.86	0–18.0	1.98	2.66	0–18.9
≥30 years	1.15	1.19	0–5.2	1.67	1.47	0–7.0	4.12	2.81	0–11.2	1.33	2.04	0–17.0	1.07	1.43	0–9.0	1.44	1.59	0–12.6
*p* value	0.153			0.06			0.173			<0.001 *			0.951			0.197		
Sex																		
Women	1.31	1.32	0–5.2	1.85	1.66	0–8.4	4.01	2.59	0–11.2	1.84	2.63	0–17.0	1.13	1.58	0–9.0	1.53	2.04	0–18.9
Men	1.16	1.23	0–5.2	1.97	1.58	0–7.0	5.45	3.31	0–11.2	2.15	2.93	0–17.0	1.71	3.28	0–18.0	1.97	2.10	0–12.6
*p* value	0.628			0.484			0.013 *			0.332			0.442			0.054		
Residence																		
Urban	1.26	1.30	0–5.2	1.90	1.68	0–8.4	4.23	2.84	0–11.2	1.93	2.67	0–17.0	1.21	2.07	0–18.0	1.63	2.15	0–18.9
Rural	1.31	1.27	0–5.2	1.64	1.23	0.1–4.2	5.14	2.72	1.2–11.2	1.90	2.91	0–13.6	1.67	2.39	0–9.0	1.80	1.41	0–6.3
*p* value	0.593			0.877			0.118			0.846			0.431			0.152		
Occupation																		
Dependent worker	1.13	1.13	0–5.2	1.80	1.58	0.1–8.4	4.13	2.76	0–11.2	1.62	2.39	0–17.0	1.18	1.54	0–9.0	1.48	1.65	0–12.6
Freelance worker	0.99	1.47	0.1–5.2	1.94	1.79	0.1–7.0	4.47	3.01	0–11.2	0.92	1.01	0–3.4	0.61	0.75	0–2.4	1.60	1.47	0–6.3
Student	1.68	1.56	0–5.2	2.24	1.91	0–8.4	4.43	2.86	0–11.2	3.38	3.51	0–17.0	1.73	3.16	0–18.0	2.06	3.10	0–18.9
Unemployed	1.04	0.82	0–2.6	1.41	1.03	0.1–2.8	6.17	4.14	1.2–11.2	0.53	0.68	0–1.5	0.80	0.89	0–2.4	2.18	0.87	0.9–2.7
Housekeeper	1.19	1.10	0–3.9	1.44	0.96	0–2.8	4.28	2.31	0.4–8.4	0.95	1.31	0–3.4	0.62	0.80	0–3.0	1.43	1.10	0–2.7
Retired	1.20	0.82	0.2–2.6	1.21	0.32	0.6–1.4	3.93	3.15	0–8.4	1.06	1.35	0–3.4	2.68	3.22	0.2–9.0	0.74	0.24	0.4–0.9
*p* value	0.207			0.923			0.803			<0.001 *			0.284			0.331		
BMI																		
Underweight	2.66	2.50	0.2–5.2	2.53	2.68	0.6–5.6	8.40	2.80	5.6–11.2	6.16	4.38	1.5–10.2	1.14	1.62	0–3.0	3.30	2.74	0.9–6.3
Normal	1.53	1.38	0–5.2	2.25	1.91	0.2–8.4	4.06	2.27	0–11.2	2.38	2.87	0–17.0	1.52	2.67	0–18.0	1.96	3.04	0–18.9
Overweight	1.26	1.31	0.1–5.2	1.74	1.46	0.1–7.0	3.98	2.72	0–11.2	1.75	2.90	0–17.0	1.22	1.77	0–9.0	1.45	1.39	0–6.3
Obesity	0.89	0.93	0–3.9	1.56	1.33	0–7.0	4.91	3.44	0–11.2	1.35	1.58	0–6.8	0.94	1.70	0–9.0	1.44	1.03	0–2.7
*p* value	0.041 *			0.245			0.069			0.021 *			0.181			0.459		

Data are presented as mean, standard deviation (SD), minimum, and maximum. Comparisons between categories were performed with Mann–Whitney U test or Kruskal–Wallis test as appropriate; (*) statistical significance <0.05.

## Data Availability

Not applicable.

## Supplementary Materials

The following supporting information can be downloaded at: https://www.mdpi.com/article/10.3390/nu15040900/s1, S1: Fiber intake questionnaire administered in native language (Spanish version). S2: Scoring sheet for the estimation of dietary fiber intake in the Chilean population. S3: Questionnaire for validation by expert judges (Spanish version).
