# Supplementary material for: Pilot Study to Estimate Dietary Fiber Intake in Adults Residing in Chile
Source: Nutrients. 2023 Feb 10;15(4):900. doi: 10.3390/nu15040900 (PMC9962499; doi:10.3390/nu15040900)
Supplement: Supplementary file 1 [file nutrients-15-00900-s001.zip › Supplementary S1.pdf]

SUPPLEMENTARY MATERIAL

SECTION S1:
Fiber intake questionnaire administered in native language (Spanish version)

Ingesta de frutas
**En promedio durante el último año ¿cuántas porciones de frutas ha consumido?**<br>Los siguientes son ejemplos de porciones:<br>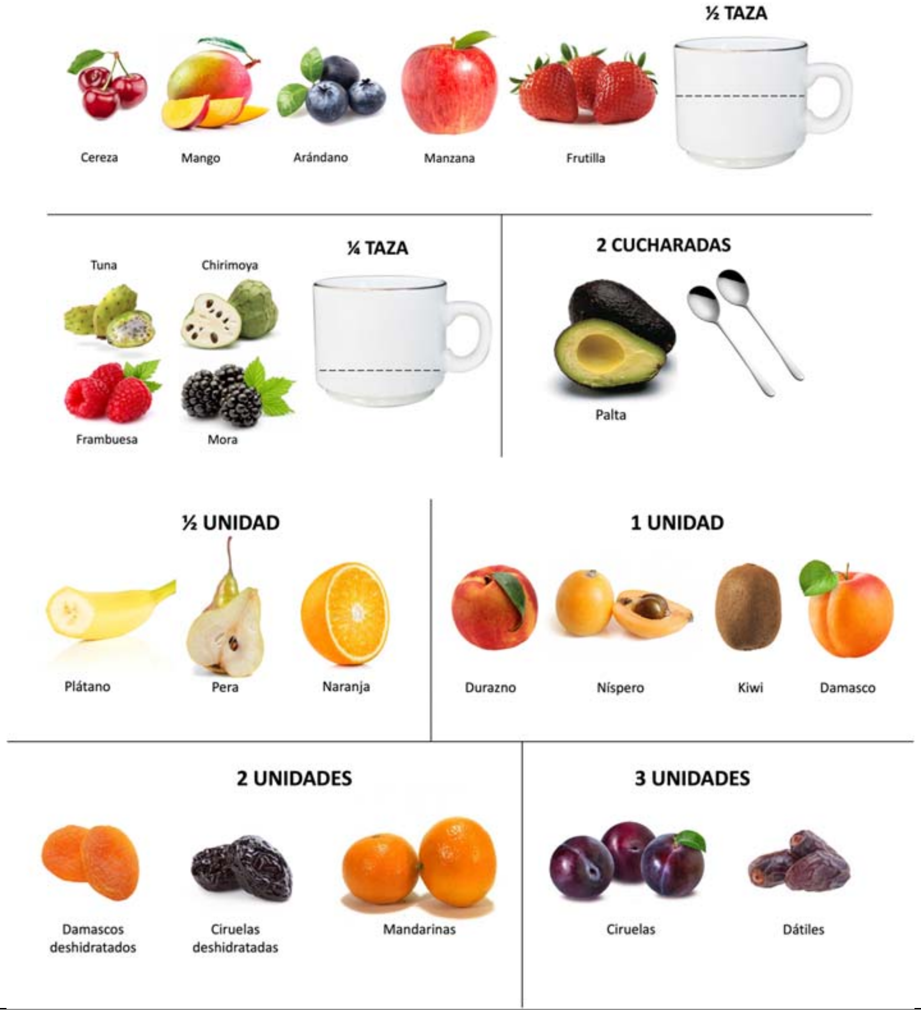

1/2 taza de: cerezas, mango, arándanos, manzana, frutilla o
1/4 taza de: tuna, chirimoya, frambuesas, moras o
2 cucharadas de palta o
1/2 unidad de plátano, pera, naranja o
1 unidad de: durazno, níspero, kiwi, damasco o
2 unidades de: damasco deshidratado, ciruelas deshidratadas, mandarinas o
3 unidades de: ciruelas, dátiles

Nunca	< de 1 porción al mes	Entre 1-3 porciones por mes	1 porción por semana	2-4 porciones por semana	5-6 porciones por semana	1 porción por día	2 porciones por día	3 porciones por día	4 porciones por día	5 porciones por día	6 o más por día

Ingesta de verduras

En promedio durante el último año ¿cuántas porciones de verduras ha consumido?
Los siguientes son ejemplos de porciones:

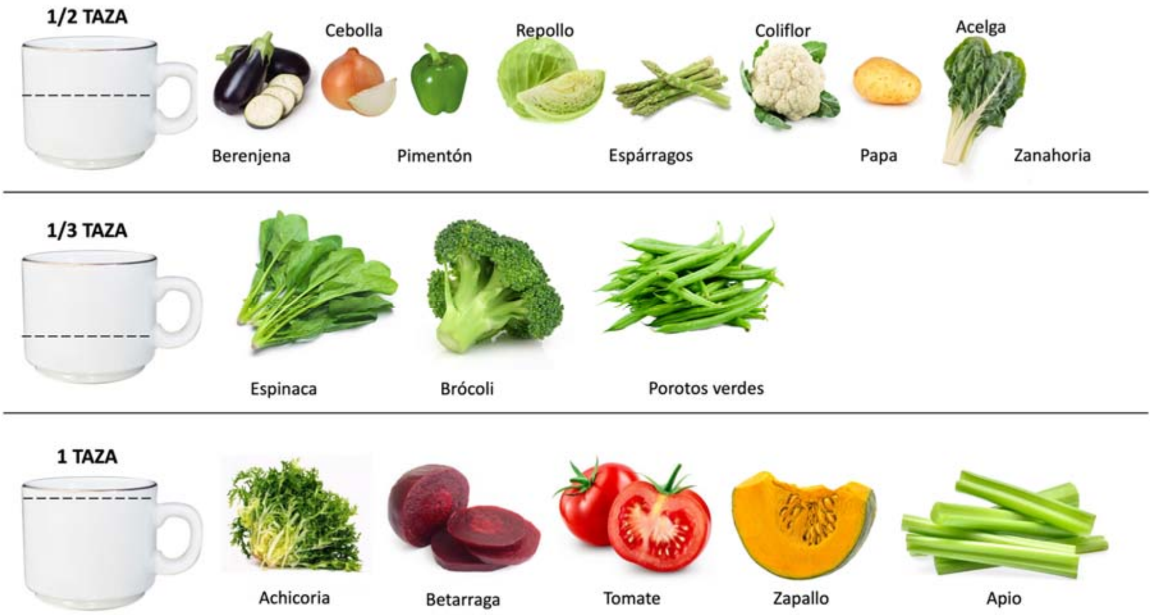

1/2 taza: berenjenas, cebolla cruda, pimiento verde, repollo crudo, espárragos, coliflor cocida, papa, acelga, zanahoria o
1/3 taza de: espinaca, brócoli, porotos verdes o
1 taza de: achicoria, betarraga, tomate crudo, zapallo camote, apio

Nunca	< de 1 porción al mes	Entre 1-3 porciones por mes	1 porción	2-4 porciones	5-6 porciones	1 porción por día	2 porciones por día	3 porciones por día	4 porciones por día	5 porciones por día	6 o más por día

			por semana	por semana	por semana						

Ingesta de pan

En promedio durante el último año ¿cuántas porciones de pan ha consumido?

Los siguientes son ejemplos de porciones:

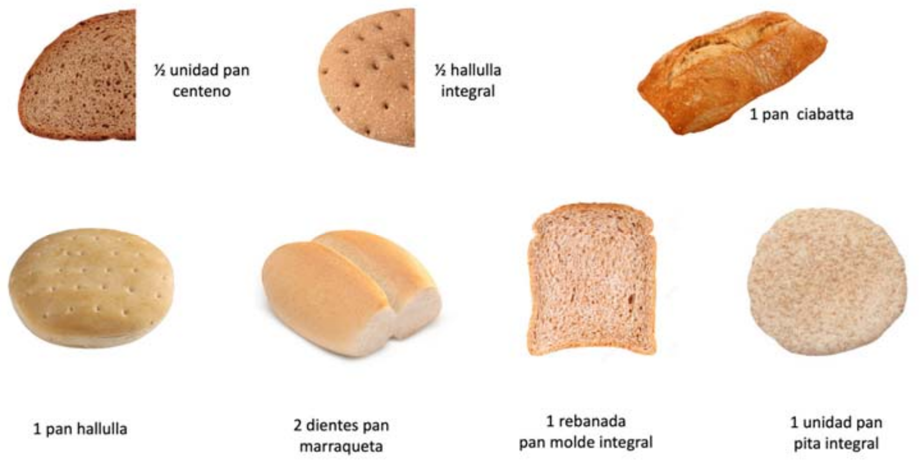

1/2 unidad de: pan centeno, hallulla integral o
1 unidad de pan ciabatta o
2 dientes de pan marraqueta o

1 rebanada de pan molde integral o
1 unidad de pan pita integral

Nunca	< de 1 porción al mes	Entre 1-3 porciones por mes	1 porción por semana	2-4 porciones por semana	5-6 porciones por semana	1 porción por día	2 porciones por día	3 porciones por día	4 porciones por día	5 porciones por día	6 o más por día

Ingesta de cereales

En promedio durante el último año ¿cuántas porciones de cereales ha consumido?
Los siguientes son ejemplos de porciones:

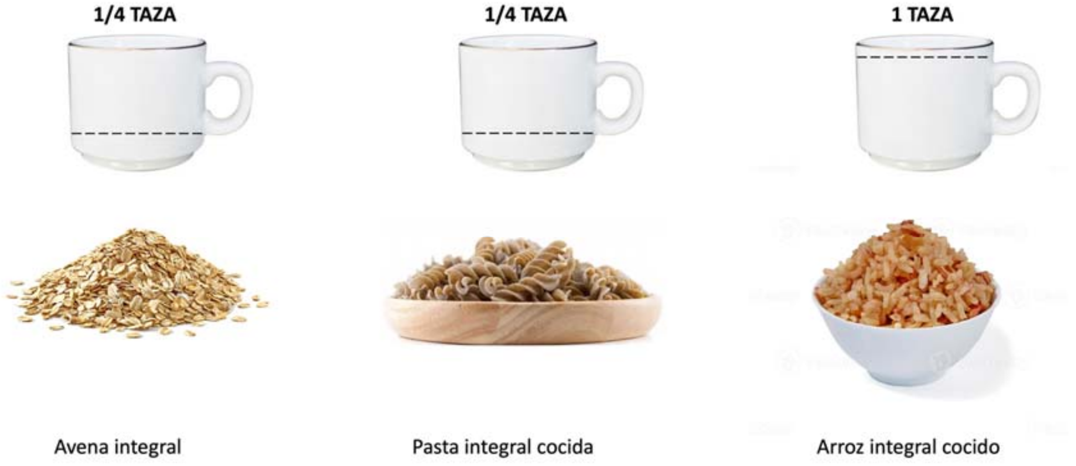

1/4 taza de: avena integral, pasta integral o
1 taza de arroz integral cocido

Nunca	< de 1 porción al mes	Entre 1-3 porciones por mes	1 porción por semana	2-4 porciones por semana	5-6 porciones por semana	1 porción por día	2 porciones por día	3 porciones por día	4 porciones por día	5 porciones por día	6 o más por día

Ingesta de frutos secos

En promedio durante el último año ¿cuántas porciones de frutos secos ha consumido?
Los siguientes son ejemplos de porciones:

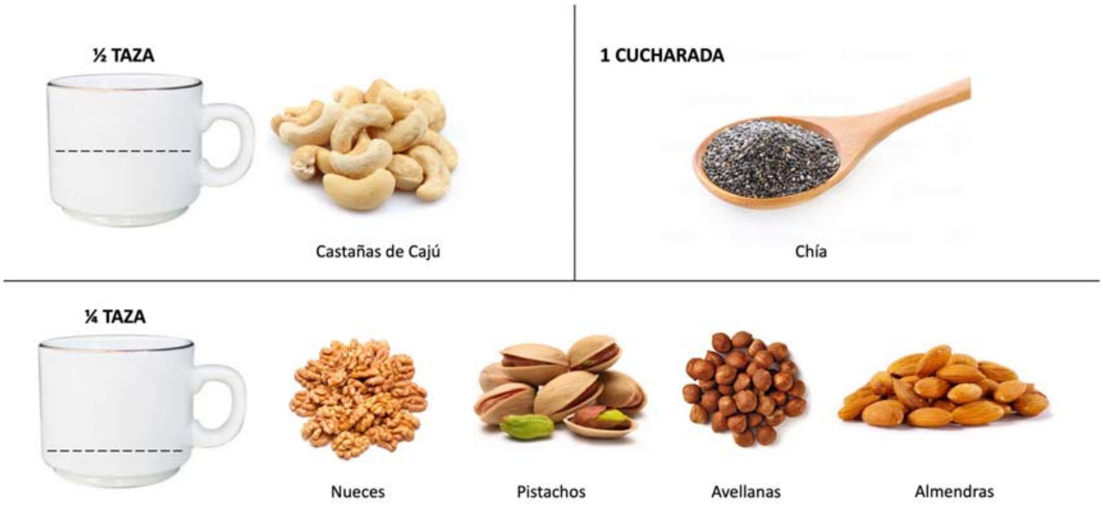

1/2 taza de: castañas de Cajú o
1/4 taza de: pistachos, avellana, nueces, almendras o
1 cucharada de chía

Nunca	< de 1 porción al mes	Entre 1-3 porciones por mes	1 porción por semana	2-4 porciones por semana	5-6 porciones por semana	1 porción por día	2 porciones por día	3 porciones por día	4 porciones por día	5 porciones por día	6 o más por día

Ingesta de legumbres

En promedio durante el último año ¿cuántas porciones de legumbres ha consumido?
Los siguientes son ejemplos de porciones:

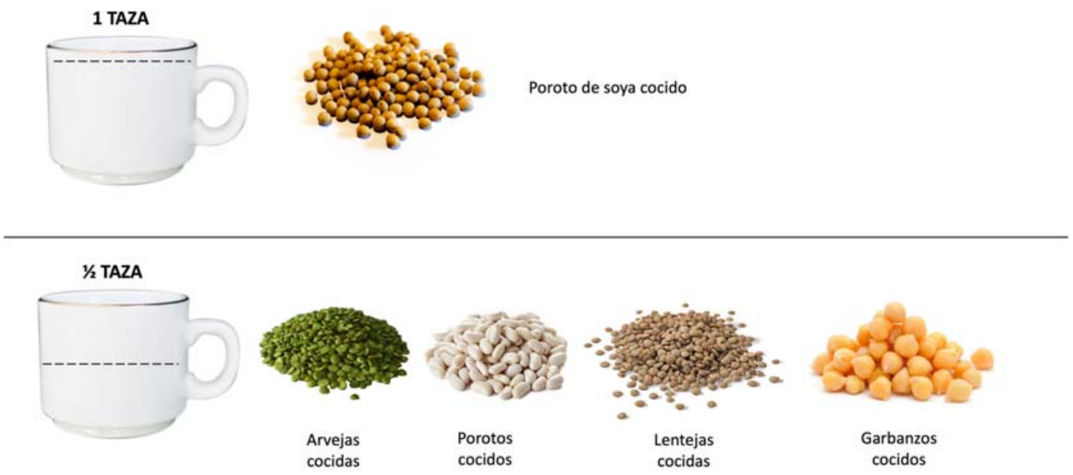

1 taza de: porotos de soya, arvejas cocidas o
1/2 taza de: porotos cocidos, lentejas cocidas, garbanzos cocidos

Nunca	< de 1 porción al mes	Entre 1-3 porciones por mes	1 porción por semana	2-4 porciones por semana	5-6 porciones por semana	1 porción por día	2 porciones por día	3 porciones por día	4 porciones por día	5 porciones por día	6 o más por día
