# Supplementary material for: Pilot Study to Estimate Dietary Fiber Intake in Adults Residing in Chile
Source: Nutrients. 2023 Feb 10;15(4):900. doi: 10.3390/nu15040900 (PMC9962499; doi:10.3390/nu15040900)
Supplement: Supplementary file 1 [file nutrients-15-00900-s001.zip › Supplementary S3.pdf]

SUPPLEMENTARY MATERIAL

SECTION S3:
Questionnaire for validation by expert judges (Spanish version)

PARTE I: INSTRUCCIONES

VALIDACIÓN POR EXPERTOS<br>"VALIDACIÓN DE UN CUESTIONARIO CORTO DE FRECUENCIA DE CONSUMO PARA FIBRA DIETARIA EN POBLACIÓN CHILENA"
PARTE I: En el siguiente documento Usted evaluará un cuestionario corto de frecuencia de consumo para fibra dietaria. Este instrumento forma parte del proyecto denominado "Propuesta para una política pública nacional de aumento de consumo de fibra dietaria en la población chilena, a través del consumo de pan". El objetivo es ejecutar un estudio exploratorio sobre el consumo de alimentos ricos en fibra en adultos chilenos, a través de un instrumento validado y autorizado para uso en esta investigación *("Validity and Reproducibility of a Habitual Dietary Fiber Intake Short Food Frequency Questionnaire". Autora: Genelle Healey*)<br><br>En cada ítem deberá hacer una evaluación, rellenando los casilleros con una X, considerando las siguientes opciones de respuesta:<br><br>1. = En total desacuerdo<br>2. = En desacuerdo<br>3. = No estoy seguro<br>4. = De acuerdo<br>5. = Totalmente de acuerdo

Posterior a cada respuesta podrá rellenar el campo “observaciones y recomendaciones”, informando sus propuestas de mejora o las dificultades de comprensión que considere. Finalizando la evaluación, se solicita contestar las hojas “Evaluación general” donde realizará una valoración general del cuestionario, e ”Identificación” donde podrá incluir sus antecedentes personales.

Ingesta de frutas

En promedio durante el último año ¿cuántas porciones de frutas ha consumido?
Los siguientes son ejemplos de porciones:

1/2 taza de: cerezas, mango, arándanos, manzana, frutilla o
1/4 taza de: tuna, chirimoya, frambuesas, moras o
2 cucharadas de palta o
1/2 unidad de plátano, pera, naranja o
1 unidad de: durazno, níspero, kiwi, damasco o
2 unidades de: damasco deshidratado, ciruelas deshidratadas, mandarinas o
3 unidades de: ciruelas, dátiles

Nunca	< de 1 porción al mes	Entre 1-3 porciones por mes	1 porción por semana	2-4 porciones por semana	5-6 porciones por semana	1 porción por día	2 porciones por día	3 porciones por día	4 porciones por día	5 porciones por día	6 o más por día

**Indique su grado de acuerdo frente a las siguientes afirmaciones.** 1= En total desacuerdo; 2= En desacuerdo; 3= No estoy seguro; 4= De acuerdo; 5= Totalmente de acuerdo	1	2	3	4	5

ADECUACIÓN					
Las porciones indicadas permiten responder con claridad a la pregunta					
La imagen permite comprender con mayor facilidad la pregunta					
Las opciones de respuesta se presentan en un orden lógico					
PERTINENCIA					
La pregunta es pertinente para lograr el objetivo del instrumento					
La imagen es pertinente para lograr el objetivo del instrumento					

Ingesta de verduras

En promedio durante el último año ¿cuántas porciones de verduras ha consumido?
Los siguientes son ejemplos de porciones:

1/2 taza: berenjenas, cebolla cruda, pimiento verde, repollo crudo, espárragos, coliflor cocida, papa, acelga, zanahoria o
1/3 taza de: espinaca, brócoli, porotos verdes o
1 taza de: achicoria, betarraga, tomate crudo, zapallo camote, apio

Nunca	< de 1 porción al mes	Entre 1-3 porciones por mes	1 porción por semana	2-4 porciones por semana	5-6 porciones por semana	1 porción por día	2 porciones por día	3 porciones por día	4 porciones por día	5 porciones por día	6 o más por día

**Indique su grado de acuerdo frente a las siguientes afirmaciones.** 1= En total desacuerdo; 2= En desacuerdo; 3= No estoy seguro; 4= De acuerdo; 5= Totalmente de acuerdo	1	2	3	4	5
ADECUACIÓN					
Las porciones indicadas permiten responder con claridad a la pregunta					
La imagen permite comprender con mayor facilidad la pregunta					
Las opciones de respuesta se presentan en un orden lógico					
PERTINENCIA					
La pregunta es pertinente para lograr el objetivo del instrumento					
La imagen es pertinente para lograr el objetivo del instrumento					

Ingesta de pan

En promedio durante el último año ¿cuántas porciones de pan ha consumido?
Los siguientes son ejemplos de porciones:

1/2 unidad de: pan centeno, hallulla integral o

1 unidad de pan ciabatta o
2 dientes de pan marraqueta o
1 rebanada de pan molde integral o
1 unidad de pan pita integral

Nunca	< de 1 porción al mes	Entre 1-3 porciones por mes	1 porción por semana	2-4 porciones por semana	5-6 porciones por semana	1 porción por día	2 porciones por día	3 porciones por día	4 porciones por día	5 porciones por día	6 o más por día

**Indique su grado de acuerdo frente a las siguientes afirmaciones.** 1= En total desacuerdo; 2= En desacuerdo; 3= No estoy seguro; 4= De acuerdo; 5= Totalmente de acuerdo	1	2	3	4	5
ADECUACIÓN					
Las porciones indicadas permiten responder con claridad a la pregunta					
La imagen permite comprender con mayor facilidad la pregunta					
Las opciones de respuesta se presentan en un orden lógico					
PERTINENCIA					
La pregunta es pertinente para lograr el objetivo del instrumento					
La imagen es pertinente para lograr el objetivo del instrumento					

Ingesta de cereales

En promedio durante el último año ¿cuántas porciones de cereales ha consumido?
Los siguientes son ejemplos de porciones:

1/4 taza de: avena integral, pasta integral o
1 taza de arroz integral cocido

Nunca	< de 1 porción al mes	Entre 1-3 porciones por mes	1 porción por semana	2-4 porciones por semana	5-6 porciones por semana	1 porción por día	2 porciones por día	3 porciones por día	4 porciones por día	5 porciones por día	6 o más por día

**Indique su grado de acuerdo frente a las siguientes afirmaciones.** 1= En total desacuerdo; 2= En desacuerdo; 3= No estoy seguro; 4= De acuerdo; 5= Totalmente de acuerdo	1	2	3	4	5
ADECUACIÓN					
Las porciones indicadas permiten responder con claridad a la pregunta					
La imagen permite comprender con mayor facilidad la pregunta					
Las opciones de respuesta se presentan en un orden lógico					
PERTINENCIA					
La pregunta es pertinente para lograr el objetivo del instrumento					
La imagen es pertinente para lograr el objetivo del instrumento					

Ingesta de frutos secos

En promedio durante el último año ¿cuántas porciones de frutos secos ha consumido?
Los siguientes son ejemplos de porciones:

1/2 taza de: castañas de Cajú o
1/4 taza de: pistachos, avellana, nueces, almendras o
1 cucharada de chía

Nunca	< de 1 porción al mes	Entre 1-3 porciones por mes	1 porción por semana	2-4 porciones por semana	5-6 porciones por semana	1 porción por día	2 porciones por día	3 porciones por día	4 porciones por día	5 porciones por día	6 o más por día

**Indique su grado de acuerdo frente a las siguientes afirmaciones.** 1= En total desacuerdo; 2= En desacuerdo; 3= No estoy seguro; 4= De acuerdo; 5= Totalmente de acuerdo	1	2	3	4	5
ADECUACIÓN					
Las porciones indicadas permiten responder con claridad a la pregunta					
La imagen permite comprender con mayor facilidad la pregunta					
Las opciones de respuesta se presentan en un orden lógico					
PERTINENCIA					
La pregunta es pertinente para lograr el objetivo del instrumento					
La imagen es pertinente para lograr el objetivo del instrumento					

Ingesta de legumbres

En promedio durante el último año ¿cuántas porciones de legumbres ha consumido?
Los siguientes son ejemplos de porciones:

1 taza de: porotos de soya, arvejas cocidas o
1/2 taza de: porotos cocidos, lentejas cocidas, garbanzos cocidos

Nunca	< de 1 porción al mes	Entre 1-3 porciones por mes	1 porción por semana	2-4 porciones por semana	5-6 porciones por semana	1 porción por día	2 porciones por día	3 porciones por día	4 porciones por día	5 porciones por día	6 o más por día

**Indique su grado de acuerdo frente a las siguientes afirmaciones.** 1= En total desacuerdo; 2= En desacuerdo; 3= No estoy seguro; 4= De acuerdo; 5= Totalmente de acuerdo	1	2	3	4	5
ADECUACIÓN					

Las porciones indicadas permiten responder con claridad a la pregunta					
La imagen permite comprender con mayor facilidad la pregunta					
Las opciones de respuesta se presentan en un orden lógico					
PERTINENCIA					
La pregunta es pertinente para lograr el objetivo del instrumento					
La imagen es pertinente para lograr el objetivo del instrumento					

PARTE II: EVALUACIÓN GENERAL

Por favor, marque con una X la respuesta:

	SÍ	NO
El instrumento contiene instrucciones claras y precisas para que un no experto en nutrición pueda responder		
Las imágenes asociadas a los listados de alimentos son apropiadas y fáciles de comprender por un no experto en nutrición		

El cuestionario es demasiado largo		

	Evaluación General del Instrumento			
	Excelente	Buena	Regular	Deficiente
Validez de contenido del instrumento				

Observaciones y recomendaciones generales del instrumento	
Motivo por el que no lo considera adecuado	
Motivo por los que no lo considera pertinente	
Propuestas de mejora	

PARTE III: IDENTIFICACIÓN DEL EXPERTO

Nombre y apellidos	
Filiación	
Correo electrónico	
Fecha de validación (dd-mm-aa)	

Muchas gracias por su valiosa contribución
